# Intramolecular bridges formed by photoswitchable click amino acids

**DOI:** 10.3762/bjoc.8.100

**Published:** 2012-06-13

**Authors:** Christian Hoppmann, Ronald Kühne, Michael Beyermann

**Affiliations:** 1 Department of Chemical Biology, Leibniz-Institut für Molekulare Pharmakologie, Robert-Rössle-Strasse 10, 13125 Berlin, Germany

**Keywords:** azobenzene, helical conformation, isomerization, molecular switches, photoswitchable click amino acid, thiol–ene click

## Abstract

Photoswitchable click amino acids (PSCaa) are amino acids bearing a side chain consisting of a photoswitchable unit elongated with a functional group that allows for a specific click reaction, such as an alkene that can react with the thiol group of a cysteine residue. An intramolecular click reaction results in the formation of a photoswitchable bridge, which can be used for controlling conformational domains in peptides and proteins. The ability to control conformations as well as the efficiency of the intramolecular bridging depends on the length of the PSCaa side chain and the distance to the cysteine residue to be clicked with. On comparing i,i+4 and i,i+7 spacings of PSCaa and cysteine in a model peptide without a preferred conformation, it was seen that the thiol–ene click reaction takes place efficiently in both cases. Upon induction of an α-helical structure by the addition of trifluoroethanol, the thiol click reaction occurs preferentially with the i,i+4 spacing. Even in the presence of glutathione as an additional thiol the click reaction of the PSCaa occurs intramolecularly with the cysteine rather than with the glutathione, indicating that the click reaction may be used even under reducing conditions occurring in living cells.

## Introduction

**Scheme 1:**
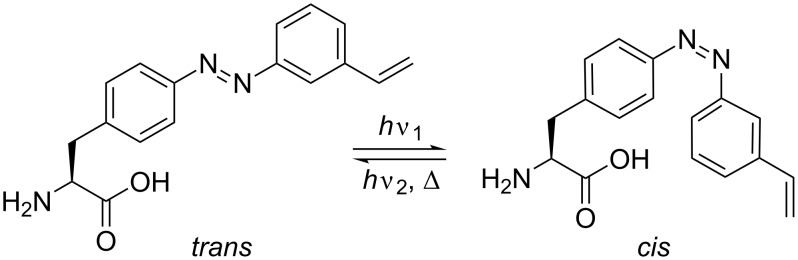
Photoisomerization of the photoswitchable click amino acid 2-amino-3-(4-((3-vinylphenyl)diazenyl)phenyl)propanoic acid.

Photoswitchable bridges that are site-specifically incorporated into proteins allow the conformation and activity of proteins to be modulated by light. In contrast to common bivalent thiol reactive azobenzene switches [1–5], the PSCaa described here (Scheme 1) is an α-amino acid containing, besides an azobenzene unit, a vinyl function that can react specifically with a cysteine within a putative conformational domain of a peptide or a protein by light-induced thiol–ene click reaction [6–9]. The light-induced click reaction of our PSCaa occurs predominantly in the *cis* state that is formed simultaneously due to the photoisomerization at λ = 365 nm.

Recently, the concept of photoswitchable click amino acids has been applied to the polypeptide hormone urocortin, the helical fold of which was regulated by light, showing different biopotencies dependent on the *trans*/*cis* isomeric state of the photocontrollable bridge [6]. In the achievement of an efficient and “clean” thiol–ene click reaction between the vinyl function of PSCaa and the cysteine residue within α-helical structures, the spacing between the two plays a pivotal role. For the i,i+4 spacing we have shown that the thiol–ene click reaction in the model peptide Ac-E-K-E-E-PSC-E-K-K-C-K-E-NH_2_ occurs smoothly without a preferred conformation, in aqueous buffered solution (pH 7.5). However, fully recombinant proteins exhibit highly structured domains, which may influence the intramolecular thiol click reaction between the PSCaa and a cysteine residue. Here, we show that the thiol click reaction of the photoswitchable click amino acid (PSCaa) at the i,i+4 position and a cysteine in a helical model peptide, under structure-inducing conditions, is favoured compared to that with PSCaa at the i,i+7 position. Furthermore, in the presence of glutathione (GSH), a naturally occurring thiol in living cells, the click reaction takes place at relatively high GSH concentrations (1 mM) indicating that the thiol click reaction occurs preferentially intramolecularly.

## Results and Discussion

### Thiol click reaction of PSCaa within α-helical conformations

**Figure 1:**
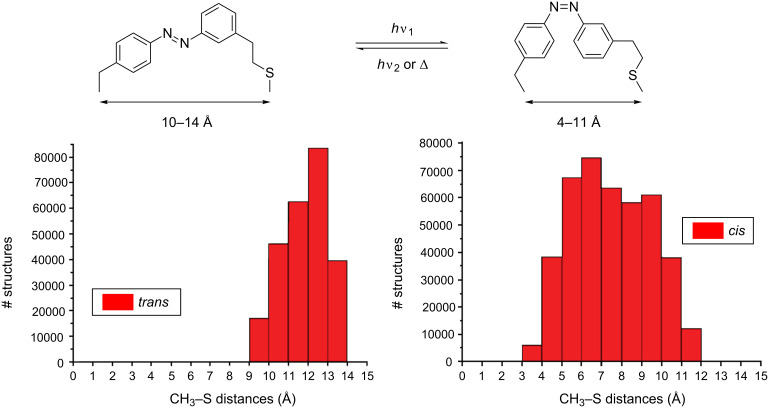
Histogram showing the distribution of end-to-end distances of the *trans* and the *cis* form between the C atom of the methyl group and cysteine S of the built-in photocontrollable bridge.

**Scheme 2:**
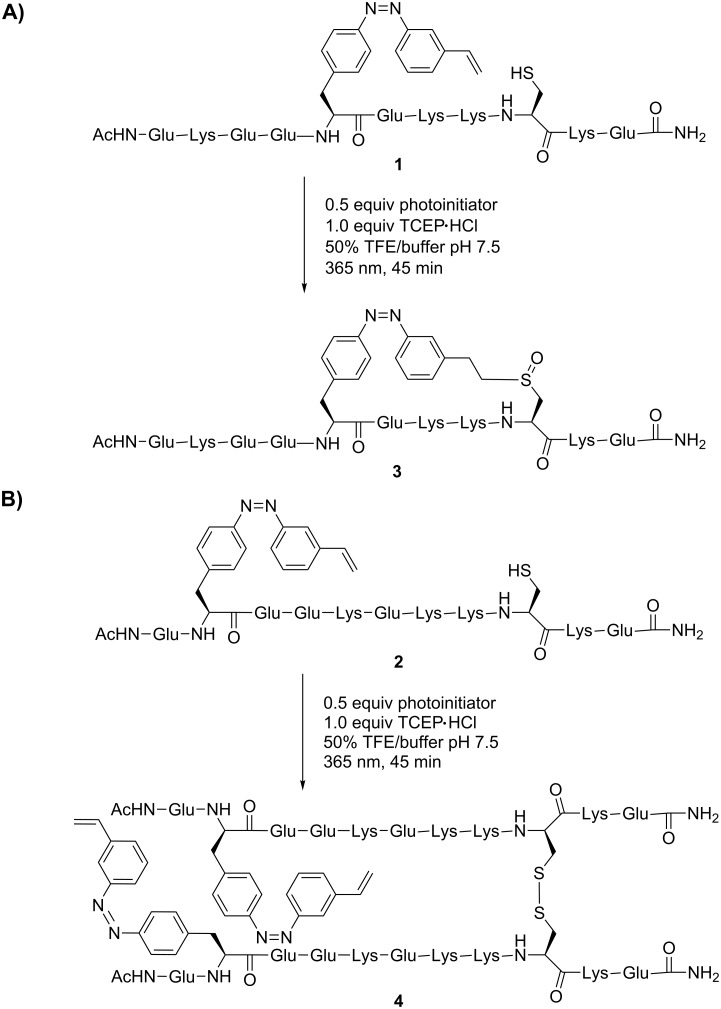
Thiol–ene click reaction of PSCaa with cysteine within the helical model peptides **1** (i,i+4) and **2** (i,i+7) under structure-inducing conditions in the presence of trifluoroethanol (50%).

Before we incorporated PSCaa into the helical model peptide at i,i+4 and i,i+7 positions, we calculated for the *trans* and the *cis* PSCaa the expected end-to-end distances between the C atom of the methyl group and the cysteine S atom of the built-in photocontrollable bridge (Figure 1). The range of distances covered by the *cis* form was found to be between 4 and 11 Å, and between 10 and 14 Å by the *trans* form. Therefore, in α-helical structures the *cis* form of PSCaa is expected to be compatible with an i,i+4 spacing (5.4 Å). Moreover, the different distances covered by the *cis* and the *trans* form of the photocontrollable bridge explain the significant stabilization of the α-helical conformation of the crosslinked peptide **3** (Scheme 2) with i,i+4 spacing in the *cis* form in contrast to the more extended *trans* form, which disturbs an α-helical conformation [6].

To study the effect of i,i+4 or i,i+7 spacings on the efficiency of intramolecular thiol–ene click reactions of the PSCaa with a cysteine in an α-helical structure, we compared the reaction in peptide **1** with i,i+4 and peptide **2** with i,i+7 spacing in the presence of 50% trifluoroethanol (TFE) (Scheme 2). The addition of TFE causes both peptides to adopt an α-helical conformation, while in aqueous buffered solution no preferred conformation of the two peptides was observed (see CD spectra in Supporting Information File 1). Interestingly, the overall helix content of peptide **2** is lower (42%) than that of peptide **1** (77%) indicating that the PSCaa positioned at i,i+7 disturbed the α-helical conformation in our model peptide. However, for the herein described investigation the induction of α-helical conformation by adding TFE is sufficient for the study of this effect.

Irradiation of the reaction mixture at λ = 365 nm for 45 min induces not only the thiol–ene click reaction but simultaneously the *trans*-to-*cis* photoisomerization. Under these conditions the formation of the intramolecular bridge resulted in click product **3** with i,i+4 helical spacing, whereas for peptide **2** with i,i+7 spacing only traces of the intramolecular click product were observed. Most notably, for peptide **2** (i,i+7) the predominate formation of the disulfide **4** was detected, indicating that the intramolecular thiol–ene click reaction within i,i+7 helical spacing is inefficient (Scheme 2). In contrast, in aqueous buffered solution, in which both peptides adopted no preferred conformation, the click products of either peptide **1** (i,i+4) or **2** (i,i+7) were obtained equally. These findings indicate that in α-helical structures, i,i+4 spacing of PSCaa and the cysteine residue preferentially allows the intramolecular thiol click reaction to take place, in contrast to the case of i,i+7 spacing.

### Thiol click reaction in presence of glutathione (GSH)

For the formation of intramolecular bridges in proteins, even under conditions in living cells, it is important that the intramolecular thiol–ene click reaction takes place in the presence of endogenous thiols. In most cells the cysteine containing tripeptide glutathione (GSH) is present at millimolar concentrations (0.1 mM–10 mM). Therefore, we investigated the thiol–ene click reaction of PCSaa and cysteine within peptide **1** and **2** in buffered solution (peptide concentration 0.1 mM) in the presence of GSH at different concentrations (10 mM, 5 mM, 1 mM, 0.5 mM, 0.1 mM).

**Figure 2:**
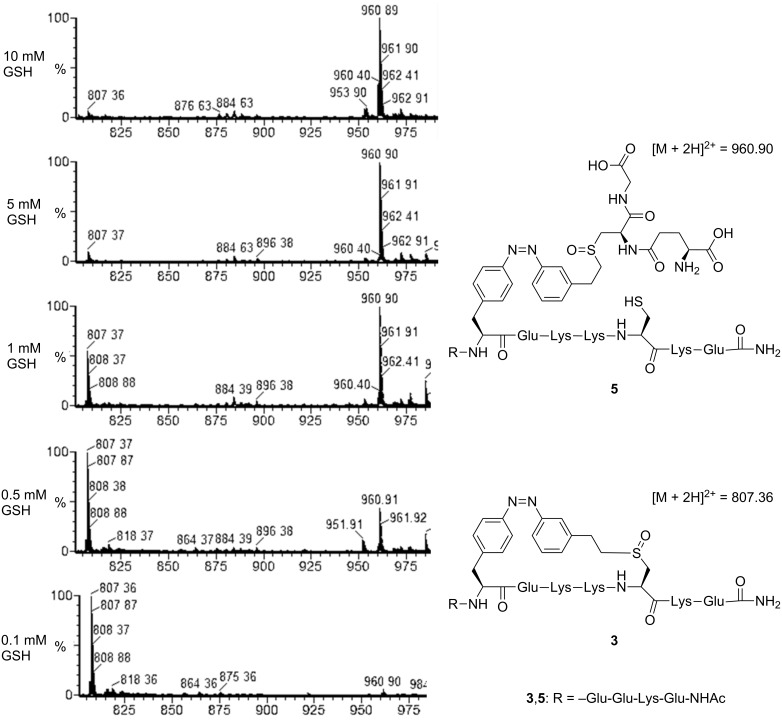
ESI–MS spectra of fractions of the crude reaction solution of the thiol–ene click reaction of peptide **1** (peptide concentration 0.1 mM) in the presence of GSH (10 mM, 5 mM, 1 mM, 0.5 mM, 0.1 mM), showing the intermolecular click product **5** at high GSH concentrations and the intramolecular click product **3** at decreasing GSH concentrations.

Simply by comparing the intensity of the mass peaks of the intramolecular with that of the intermolecular click product, we found that with decreasing GSH concentration the yield of the intramolecular thiol click reaction increased (Figure 2, data shown for peptide **1**). At the highest GSH concentration tested (10 mM), only the intermolecular click product **5** ([M + 2H]^2+^ = 960.90) was detected as the corresponding sulfoxide of the thioether formed in the crosslinked peptide **3**. Already, in the presence of 1 mM GSH the intramolecular thiol click products ([M + 2H]^2+^ = 807.36) of both peptides with i,i+4 and i,i+7 spacings were obtained (for ESI–MS spectra of peptide **2** with i,i+7, see Supporting Information File 1). Our results show that the intramolecular thiol–ene click reaction of PSCaa and cysteine is preferred compared to the intermolecular reaction of PSCaa with glutathione, providing perspectives for an intracellular application of this reaction type within recombinant proteins.

## Conclusion

The light-induced thiol–ene click reaction of our PSCaa with cysteine occurs in its *cis* form, which is predominantly formed under the conditions of the inducing light (λ = 365 nm). The distribution of calculated end-to-end distances of the *cis* form (4–11 Å) is compatible with the i,i+4 spacing (5.4 Å) in a helical peptide. Accordingly, the intramolecular click reaction with i,i+4 spacing occurs more efficiently when a helical conformation of the model peptide has been stabilized by the addition of TFE rather than without a preferred conformation in the absence of TFE. Without a preferred peptide conformation, the click reaction proceeds smoothly with both i,i+4 and i,i+7 spacings. Our results indicate that our PSCaa incorporated into helical domains of proteins may allow the formation of photoswitchable bridges for controlling the conformation of biologically important protein domains. The intramolecular click reaction takes place even under reducing conditions, thus lending itself to an application in vivo in combination with protein synthesis with ad hoc evolved orthogonal tRNA/synthease pairs in an ongoing project [10].

## Experimental

The photoswitchable click amino acid 2-amino-3-(4-((3-vinylphenyl)diazenyl)phenyl)propanoic acid (PSCaa) and the helical model peptides were synthesized as described in [6]. Thiol–ene coupling was performed at a peptide concentration of 0.1 mM in the presence of the photoinitiator 2-hydroxy-1-[4-(2-hydroxyethoxy)phenyl]-2-methyl-1-propanone (0.5 equiv) and TCEP·HCl (1.0 equiv) in degassed buffered solution containing 50% trifluoroethanol (pH 7.5). The reaction mixture was exposed to λ = 365 (4 mW/cm^2^) for 45 min. LC–MS analysis was performed on an ACQUITY UPLC system equipped with a C18 column (3 μm, 2.1 × 30 mm) in combination with an electrospray time-of-flight (ESI–TOF) mass spectrometer (LCT Premier) from Waters. LC conditions: flow 0.2 mL/min, temperature 30 °C, eluent systems: eluent A = 1% acetonitrile in water (0.05% TFA), eluent B = 99% acetonitrile in water (0.05% TFA), linear gradient of 5 to 95% B in 6 min. UV detection was performed at 220 and 358 nm. CD spectra were recorded on a JASCO spectrophotometer (J-720) in a quartz cell of 0.1 cm path length over the range 198–300 nm at 25 °C. Peptide concentration was ~1 × 10^−4^ M in phosphate buffer pH 7.5. Obtained CD spectra were the average of six accumulations made at 0.1 nm intervals, reported in terms of molar ellipticity per residue ([θ]_r_) in deg × cm^2^ × dmol^−1^. The calculation of end-to-end distance changes for each isomer was realized with the Tripos FF method [11–13]. For each isomer 250,000 structures were generated maintaining a threshold of 5 kcal/mol.

## Supporting Information

File 1 CD spectra of **1** and **2** and ESI–MS spectra of peptide **2** in the presence of GSH.
