# Supplementary material for: Intramolecular bridges formed by photoswitchable click amino acids
Source: Beilstein J Org Chem. 2012 Jun 13;8:884–9. doi: 10.3762/bjoc.8.100 (PMC3388878; doi:10.3762/bjoc.8.100)
Supplement: File 1 — CD spectra of 1 and 2 and ESI–MS spectra of peptide 2 in the presence of GSH. [file Beilstein_J_Org_Chem-08-884-s001.pdf]

Supporting Information

for

Intramolecular bridges formed by photoswitchable click amino acids

Christian Hoppmann*, Ronald Kühne and Michael Beyermann

Address: Department of Chemical Biology, Leibniz-Institut für Molekulare Pharmakologie, Robert-Rössle-Strasse 10, 13125 Berlin

Email: Christian Hoppmann - hoppmann@fmp-berlin.de

* Corresponding author

CD spectra of **1** and **2** and ESI–MS spectra of peptide **2** in the presence of GSH

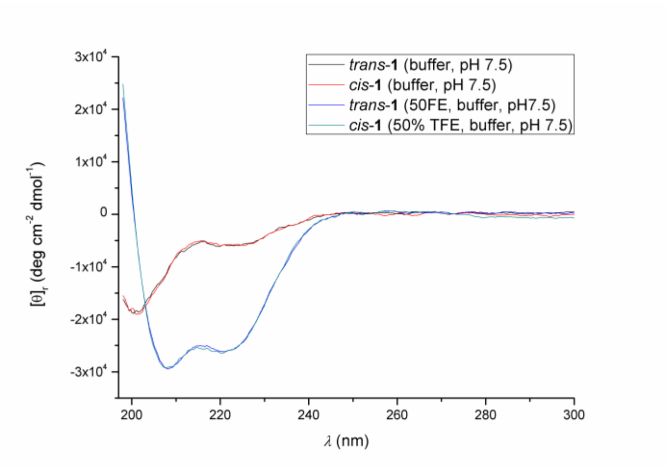

Figure S1: Circular dichroism spectra of peptide **1** in buffered solution and 50% TFE.

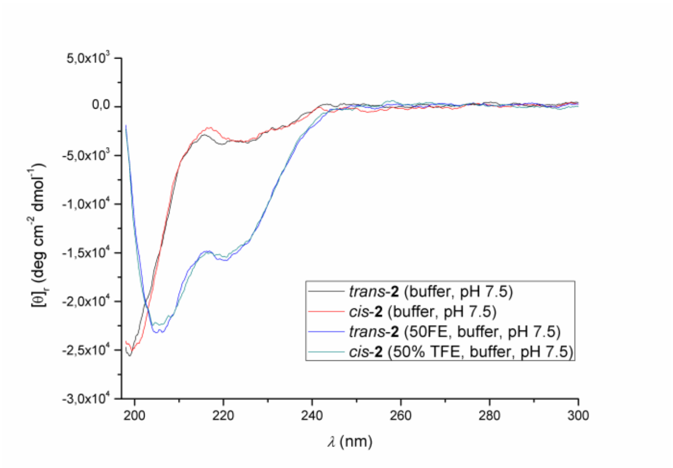

Figure S2: Circular dichroism spectra of peptide **2** in buffered solution and 50% TFE.

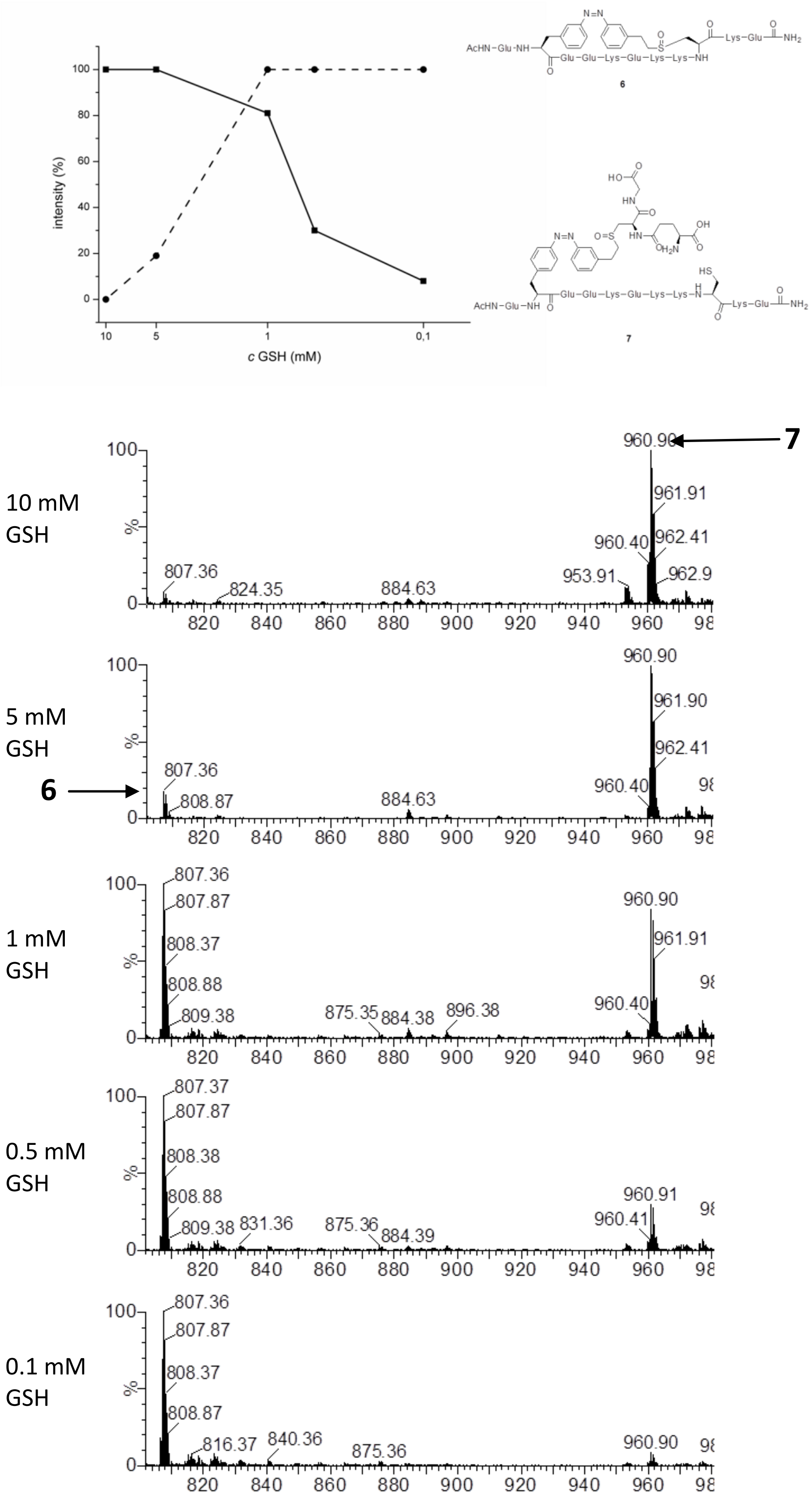

Figure S3: Intramolecular vs intermolecular thiol click reaction of peptide **2** with i,i+7 helical spacing.
